# Supplementary material for: Deletion of wfs1 Impairs Oligodendrocyte Precursor Cells Dorsal Distribution and Myelination Through the wfs1-hmgcs1 Axis in Zebrafish
Source: Biology (Basel). 2026 Aug 16;15(16):1402. doi: 10.3390/biology15161402 (PMC13510151; doi:10.3390/biology15161402)
Supplement: Supplementary file 1 [file biology-15-01402-s001.zip › File S1-Transcriptome sequencing data and differentially expressed genes (DEGs) in wfs1-- zebrafish at 5 dpf/2.KEGG/wfs1KOvsWT/DOWN/src/dre00270.html]

dre00270


- dre:550524

- Down regulated genes

550524\_1(-2.57926437647488)

- dre:571201

- Down regulated genes

571201(-9.03201015725177)

- dre:334195

- Down regulated genes

334195(-1.02556051088404)
- dre:678634

- Down regulated genes

678634(-2.56566805889366)

- dre:791167

- Down regulated genes

791167(-8.30993863819111)

- dre:431773

- Down regulated genes

431773(-4.19518690776961)

- dre:406688

- Down regulated genes

406688(-1.12341806422973)

- dre:406688

- Down regulated genes

406688(-1.12341806422973)

- dre:571201

- Down regulated genes

571201(-9.03201015725177)

- dre:406688

- Down regulated genes

406688(-1.12341806422973)

- dre:571201

- Down regulated genes

571201(-9.03201015725177)

Close
